# Overground walking with a robotic exoskeleton elicits trunk muscle activity in people with high-thoracic motor-complete spinal cord injury

**DOI:** 10.1186/s12984-018-0453-0

**Published:** 2018-11-20

**Authors:** Raed A. Alamro, Amanda E. Chisholm, Alison M. M. Williams, Mark G. Carpenter, Tania Lam

**Affiliations:** 1 School of Kinesiology, University of British Columbia, Vancouver, Canada; 2 International Collaboration on Repair Discoveries (ICORD), Blusson Spinal Cord Centre, 818 West 10th Ave, Vancouver, BC V5Z 1M9 Canada; 3 Current address: Rehabilitation Hospital, King Fahad Medical City, Riyadh Saudi Arabia

**Keywords:** Exoskeletons, Spinal cord injury, Trunk muscles, EMG

## Abstract

**Background:**

The trunk muscles are critical for postural control. Recent neurophysiological studies have revealed sparing of trunk muscle function in individuals with spinal cord injury (SCI) classified with thoracic or cervical motor-complete injuries. These findings raise the possibility for recruiting and retraining this spared trunk function through rehabilitation. Robotic gait training devices may provide a means to promote trunk muscle activation. Thus, the objective of this study was to characterize and compare the activation of the trunk muscles during walking with two robotic gait training devices (Ekso and Lokomat) in people with high thoracic motor-complete SCI.

**Methods:**

Participants with chronic motor-complete paraplegia performed 3 speed-matched walking conditions: Lokomat-assisted walking, Ekso-assisted walking overground, and Ekso-assisted walking on a treadmill. Surface electromyography (EMG) signals were recorded bilaterally from the rectus abdominis (RA), external oblique (EO), and erector spinae (ES) muscles.

**Results:**

Greater recruitment of trunk muscle EMG was elicited with Ekso-assisted walking compared to the Lokomat. Similar levels of trunk EMG activation were observed between Ekso overground and Ekso on the treadmill, indicating that differences between Ekso and Lokomat could not be attributed to the use of a hand-held gait aid. The level of trunk EMG activation during Lokomat walking was not different than that recorded during quiescent supine lying.

**Conclusions:**

Ekso-assisted walking elicits greater activation of trunk muscles compared to Lokomat-assisted walking, even after controlling for the use of hand-held assistive devices. The requirement of the Ekso for lateral weight-shifting in order to activate each step could lead to better postural muscle activation.

**Electronic supplementary material:**

The online version of this article (10.1186/s12984-018-0453-0) contains supplementary material, which is available to authorized users.

## Background

The ability to maintain postural stability during sitting or standing depends critically on motor function in the trunk muscles [1–3]. A spinal cord injury (SCI) could result in varying degrees of paralysis in these muscles, leading to deficits in postural control and balance [4]. This is thought to be especially true for those with complete injuries at or above the mid-thoracic level [5]. However, recent studies using electromyography (EMG), ultrasound, or transcranial magnetic stimulation have revealed sparing of trunk muscle function in people with high-thoracic motor-complete SCI [6–8]. These findings open up the possibility for developing techniques to recruit and train this preserved function.

In able-bodied individuals, the trunk muscles are rhythmically activated during walking to maintain upper body steadiness [9, 10], and the amplitude of trunk muscle activity increases with increasing gait speed as a result of the higher postural stability demands [11, 12]. Robotic exoskeletons, such as the Lokomat® and Ekso™, are used to facilitate gait training for people with SCI [13–16], but it remains unknown the extent to which they engage those trunk muscles that are normally activated during walking. The Lokomat provides gait training on a treadmill with the trunk passively supported by an overhead harness with varying levels of body weight support, depending on the functional status of the subject. Although modulating the level of body weight support has been touted as a key factor in facilitating locomotor recovery, it also implies lesser degree of recruitment of postural muscles (since the body is supported and held rigidly within the Lokomat). In contrast, gait training with the Ekso is provided overground with the assistance of a hand-held device (wheeled walker or forearm crutches) and requires active participation from the user to shift their center of mass forward and laterally in order to trigger the initiation of each step. It should be expected, therefore, that using the Ekso could better facilitate trunk muscle recruitment, and ultimately, postural control.

The purpose of this study was to characterize and compare the activation patterns of the trunk (abdominal and paraspinal) muscles during walking with two different robotic exoskeletons (Lokomat and Ekso) in people with high thoracic motor-complete SCI. A secondary aim was to reference the activation patterns of the trunk muscles elicited from these two exoskeletons in people with SCI with those normally observed during regular overground walking in able-bodied individuals. We hypothesized that Ekso-assisted overground walking will elicit greater trunk muscle activation compared to Lokomat-assisted walking, even after controlling for the effects of the hand-held assistive device.

## Methods

### Participants

Participants with SCI were recruited to this study. Inclusion criteria were 19–65 years of age, chronic (> 1 year) motor-complete SCI (American Spinal Injury Association Impairment Scale; AIS A and B), and injury level between C7-T6. Able-bodied participants were also recruited, with the same inclusion criteria listed above except for history of spinal cord injury. All subjects had to be within the capacity limits of the Lokomat and Ekso (weight < 100 kg, height between 157 and 188 cm, standing hip width < 47.5 cm, and near normal range of motion in hips, knees and ankles) and in stable medical condition. They should have also received familiarization training to walk with the Ekso and Lokomat and achieved a walking speed of at least 1 km/h. All study procedures were approved by the UBC Clinical Research Ethics Board and all participants provided their voluntary, written consent to participate in the study.

### Procedures

#### Assessment of impairment

Participants with SCI were evaluated and classified as per the International Standards for Neurological Classification of Spinal Cord Injury (ISNCSCI) [5] by an experienced registered nurse working in a spinal cord injury unit.

#### EMG recordings

Surface electromyography (SX-230-1000 (active EMG probes), Biometrics Ltd., Newport, UK) was used to record EMG activity of the following muscles, bilaterally: rectus abdominis (RA), external oblique (EO) and erector spinae (ES). The EMG electrodes were taped to the skin as follows: RA – 3 cm lateral and 2 cm caudal to umbilicus; EO – 2 cm below the lowest point of the rib cage; ES – 2 cm lateral to the L4-L5 spinous processes. Prior to attaching the EMG electrodes, the skin area was prepared by shaving and cleansing the site using alcohol swabs to reduce skin impedance. To minimize movement artifact and maintain a stable recording site, we wrapped a wide tensor cloth around the trunk under participants’ clothing to further secure the electrodes in place.

To normalize the EMG amplitude, participants were asked to perform (or attempt) voluntary maximum contractions (MVC) while lying supine on a plinth. The contraction was timed to exhalation, such that for the RA, participants were instructed to first exhale for 2 s, inhale in (2 s), and then exhale while attempting trunk flexion and hold for 5 s [8]. Following the same breathing and stabilization procedures, the MVC of the EO was recorded while the participants attempted lateral trunk flexion to the right and to the left. For the ES MVC, the participants attempted back extension while lying prone. Each MVC trial was repeated twice with a 1-min rest between attempts. A research assistant used his hands to stabilize participants against the plinth by the shoulders. Periods of quiescent baseline EMG activity (BAS) for each muscle were also recorded while participants lay relaxed on the plinth. Breathing was monitored by the change in air temperature detected by a custom-made thermocouple placed under the participants’ right or left naris, with warmer temperature indicating exhalation and cooler temperature indicating inspiration.

#### Walking trials

Participants were provided familiarization training and data collection for this study was initiated as soon as they achieved a minimum overground walking speed of 1 km/h. EMG signals were recorded while participants performed three walking conditions: 1) Ekso overground (Ekso-OG); 2) Ekso on a treadmill (Ekso-TM); and 3) Lokomat (Loko-TM). All trials were recorded in a single session and the order of the walking conditions was quasi-randomized such that the Loko-TM was always either the first or last condition completed. We used foot switches (FS4, Biometrics, Newport, UK) placed under the heel and toe of each foot to determine heel strike and toe off for each step. An accelerometer (ACL300, Biometrics, Newport, UK) was affixed over the spinous process of C7 to detect the trunk acceleration. Data recorded from the foot switches and accelerometer along with the EMG and thermocouple were sampled at 1000 Hz through a portable data acquisition system (DataLOG, Biometrics, Newport, UK). EMG and kinematic data were recorded for at least 60 strides. Rest breaks were provided as needed.

##### Ekso-OG trial

Participants walked back and forth along a 14-m walkway at their comfortable speed and with the aid of a four-wheeled walker. Speed was calculated from the time taken to traverse the middle 10 m of the walkway, as measured by a stopwatch. All participants walked with the “ProStep” mode, in which steps are automatically triggered when the Ekso sensed the participant reached weight-shifting targets.

##### Ekso-TM trial

To control for the effect of hand-held gait aid required of Ekso-OG walking, participants also walked with the Ekso on a treadmill and were instructed to use the treadmill’s side handrails for support. The Ekso was attached to an overhead tether for safety. Treadmill speed was matched to that during the Ekso-OG trial.

##### Loko-TM trial

Participants walked with the Lokomat and provided approximately 50% body weight support, which was required to ensure proper stance limb kinematics (i.e. upright posture, hip and knee joints extended) during walking. Participants were instructed to use the treadmill’s side handrails for support, and the treadmill speed was matched to the other walking conditions. To determine the effects of body weight support (BWS) on trunk muscle activity, able-bodied participants performed an additional Loko-TM condition with 5 kg BWS (the lowest BWS possible) to be compared with their regular 50% BWS trial (as required by the SCI subjects during Loko-TM condition).

For reference data, able-bodied participants also completed a trial of 60 strides of regular treadmill walking at matched speeds to the walking trials recorded from the SCI participants.

#### Data analysis

All EMG data were high-pass filtered with a 6th-order dual pass Butterworth filter at 30 Hz, rectified, then low-pass filtered at 50 Hz using custom-written routines in MATLAB (Mathworks, Natick, MA, USA). For each MVC trial, EMG amplitude was calculated by calculating the root mean square (RMS) of the middle 1000-ms period of the trial. The average RMS value of the two MVC trials was then calculated and used to normalize the amplitude of the EMG signal obtained during the walking trials.

The filtered and rectified EMG data of the walking trials were divided into gait cycles using signals from the footswitches (right heel strike to the next right heel strike). The RMS amplitude was calculated over each gait cycle for each muscle in each condition. The mean RMS across all gait cycles in each condition was then calculated for each muscle and normalized to that muscle’s MVC to represent EMG amplitude as a percentage of MVC. Then the RMS values for the right and left sides were summed for each muscle for each participant.

Accelerometer and breathing data were filtered with a fourth-order dual pass Butterworth filter at a low-pass of 1 Hz before it was divided into individual periods (gait cycles) to show breathing activity and trunk acceleration during walking. To quantify trunk movement across the different walking conditions, the minimum trunk anterior-posterior and medial-lateral accelerations were subtracted from their maximum in each direction for each walking condition and then averaged for each subject. To determine whether any rhythmic activation of the trunk muscles could be linked to breathing, the trunk muscle EMG data was also divided according to the respiratory rhythm, synchronized to the onset of inhalation.

Data from the able-bodied participants were recorded and analyzed in the same way to provide a reference and for descriptive purposes.

#### Statistical analysis

Statistical tests were performed on the SCI data only and analyzed at an alpha of 0.05 using SPSS v.20 (IBM Inc., Armonk NY). EMG amplitude of each muscle was compared across 4 conditions: BAS, Loko-TM, Ekso-TM, and Ekso-OG. Trunk acceleration was compared across 3 walking conditions (Loko-TM, Ekso-TM, and Ekso-OG). All comparisons were performed using a repeated measure ANOVA. Four a priori post-hoc comparisons were performed to determine the effect of each robotic device compared to quiescent baseline activity (BAS vs. Loko-TM and BAS vs. Ekso-TM), the difference between devices (Loko-TM vs. Ekso-TM), and the effect of hand-held gait aid (Ekso-TM vs. Ekso-OG). The Bonferroni correction was used to account for the 4 multiple comparisons (adjusted alpha = 0.05/4 = 0.0125). For trunk acceleration, 2 a priori post-hoc comparisons were planned to compare between Loko-TM vs. Ekso-TM and Ekso-OG vs. Ekso-TM (adjusted alpha = 0.05/2 = 0.025). For statistically significant results, partial Eta squared was calculated to report effect size. Observed power was also reported. Prior to conducting ANOVA, normality of the data was checked by Shapiro-Wilk test. The assumption of Sphericity was tested with Mauchly’s test, and when violated, Greenhouse-Geisser correction was applied. EMG amplitude of each muscle was also compared between Loko-TM with 5 kg BWS vs. 50% BWS with a paired t-test in able-bodied participants.

## Results

### Participant characteristics

Eight SCI and eight able-bodied subjects participated in this study. The SCI subjects were able to complete all assessment procedures and the three testing conditions with walking speeds ranging from 1.0 to 1.4 km/h. Data collection was initiated after SCI subjects could achieve an overground walking speed with the Ekso of at least 1.0 km/h; this took no more than 5 familiarization sessions across all participants. The SCI participant characteristics are summarized in Table 1. Able-bodied individuals were four males and four females, with age ranging between 20 and 41 years (mean: 28 years), height range: 167.5–179 cm (mean: 171.12 cm), weight range: 55–83.9 kg (mean: 66.88 kg) and tested walking speed ranging between 1 and 1.4 km/h (mean: 1.2 km/h).

**Table 1:** Detailed characteristics of participants with SCI

Subject ID	Age (yrs)	Sex	Height (cm)	Weight (kg)	Years post-injury	Level of Injury	AIS	Sensory score		UEMS	ZPP (R/L)	Testing speed (km/h)
								Light touch	Pin-prick			
S01	33	M	177	68	13	T4	A	48	49	50	T6/T6	1.1
S02	41	M	183	92	23	T3	A	41	43	50	T3/T5	1.3
S03	42	M	170	68	19	C7	B	66	68	34	–	1.3
S04	39	F	177	69	25	T3	A	44	42	50	T4/T4	1.4
S05	32	M	191	100	7	T4	A	46	50	50	T6/T6	1.0
S06	36	M	178	79	1	C7	A	27	28	29	T2/T1	1.0
S07	32	M	175	79	3	C7	B	42	44	50	–	1.1
S08	52	M	177	75	2	T4	A	46	46	50	T5/T5	1.1

Abbreviations: *M* = male, *F* = female, *UEMS* = Upper Extremity Motor Score, *AIS* = American Spinal Injury Association Impairment Scale, *A* = complete impairment, *B* = sensory incomplete, *ZPP R/L* = zone of partial preservation on the right and left side

### Maximum voluntary contraction

**Fig. 1:**
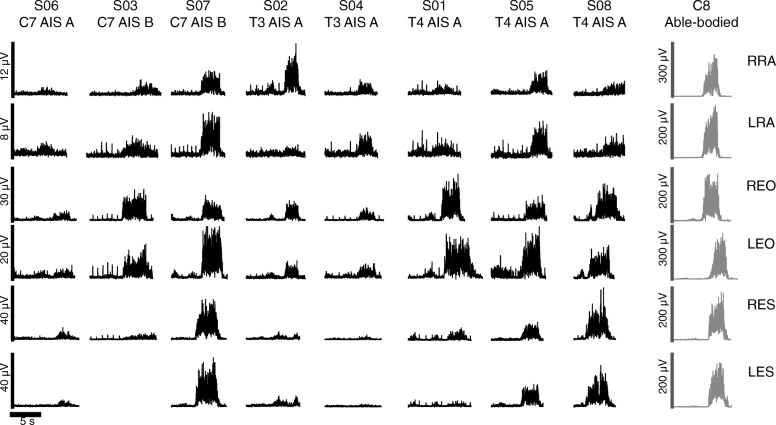
Trunk muscle EMG activity during maximum voluntary contractions. Filtered and rectified EMG activity recorded from the trunk muscles during the maximum voluntary contraction (MVC) trials in each SCI subject (black) and a representative able-bodied subject (C8) (grey). SCI subjects are plotted in sequence from left to right according to their injury level from highest to lowest. R/L = right/left, RA = Rectus Abdominis, EO = External Oblique, and ES = Erector Spinae. (Left ES data was not available from subject S03 due to technical error)

Figure 1 shows trunk muscle activity during MVC trials in each SCI subject and a representative able-bodied subject. Although clinically classified as motor-complete, all SCI subjects exhibited the ability to voluntarily recruit at least one of the recorded trunk muscles.

### Trunk muscle activation patterns during robotic-assisted walking

**Fig. 2:**
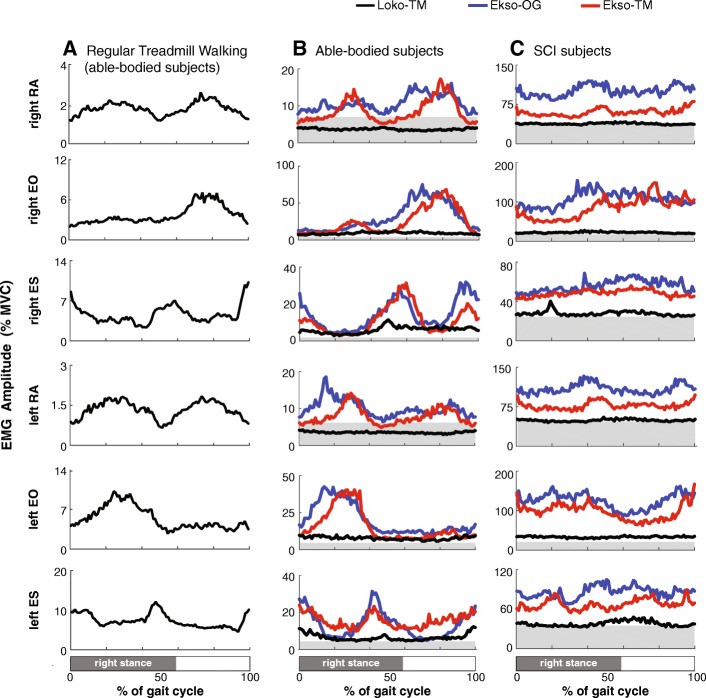
Trunk muscle activation patterns during robotic-assisted walking in able-bodied and SCI subjects. **a**) Mean trunk muscle activity patterns averaged across able-bodied participants during walking on treadmill with an average speed of 1.2 km/h. **b**) Mean trunk muscle activity patterns averaged across able-bodied participants during walking in the Lokomat (Loko-TM), Ekso on treadmill (Ekso-TM) and Ekso overground (Ekso-OG) with an average matched speed across conditions of 1.26 km/h. All plots represent the mean trunk muscle activity normalized to 100% of the gait cycle (*n* > 20 steps each plot for each subject). Grey shaded areas in each plot represent baseline EMG activity recorded in supine position (BAS). **c**) Mean trunk muscle activity patterns averaged across all SCI subjects during the same walking conditions with an average matched speed across conditions of 1.16 km/h. RA = Rectus Abdominis, EO = External Oblique, and ES = Erector Spinae

Figure 2 shows ensemble EMG gait patterns during normal treadmill walking (Fig. 2a) and robotic-assisted walking (Fig. 2b) from able-bodied subjects compared to the ensemble averaged EMG data from the SCI subjects during robotic-assisted walking (Fig. 2c). Individual subject data are provided in Additional file 1: Figure S1. The trunk muscles showed higher muscle activity during the Ekso walking conditions (Ekso-OG and Ekso-TM) compared to Loko-TM in both the SCI and the able-bodied subjects. Moreover, trunk muscle activation patterns during Ekso-assisted walking were comparable to EMG patterns during regular treadmill walking in able-bodied individuals at similar speeds.

### EMG amplitude

**Fig. 3:**
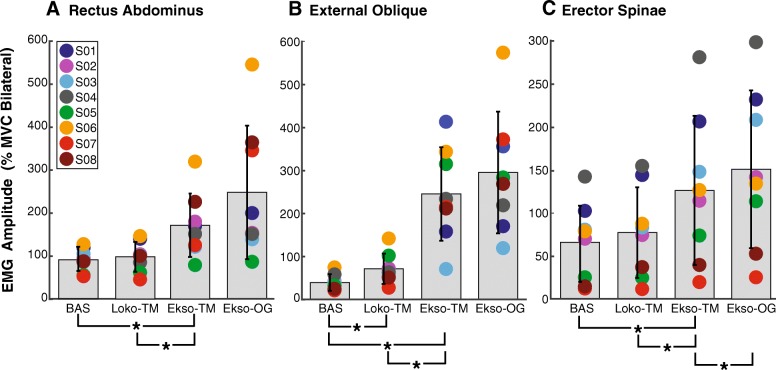
Trunk muscle EMG amplitudes across baseline and walking conditions. Comparison of EMG amplitude recorded during quiet supine lying and robot-assisted walking conditions. The average RMS EMG amplitude in rectus abdominis (**a**) external oblique (**b**), and erector spinae (**c**) during quiet supine lying (BAS), Lokomat-assisted walking (Loko-TM), Ekso on the treadmill (Ekso-TM) and Ekso overground walking (Ekso-OG) are plotted for each SCI participant (represented by different coloured circles). Grey bars represent the RMS EMG amplitude averaged across all SCI participants and error bars represent the standard deviation. Values from left and right homologous muscles were expressed as % MVC and summed bilaterally. * = *p* < 0.0125

There was a main effect of walking condition in all muscles (RA: F_1.09, 7.60_ = 8.57, *p* = 0.019, partial η^2^ = 0.55, observed power = 0.74; EO: F_1.68, 11.74_ = 24.72, *p* < 0.001, partial η^2^ = 0.78, observed power = 1.000; ES: F_1.25, 8.74_ = 18.17, *p* = 0.002, partial η^2^ = 0.72, observed power = 0.98) (Fig. 3). Pairwise post-hoc comparisons revealed that Loko-TM elicited greater activation only in EO (*p* = 0.008), but not in RA (*p* = 0.112) or ES (*p* = 0.062) compared to baseline quiescent activity. However, Ekso-TM elicited significantly greater activation in RA (*p* = 0.006), EO (*p* = 0.001), and ES (*p* = 0.005) compared to baseline. EMG amplitude in all muscles was also significantly greater in Ekso-TM compared to Loko-TM (RA: p = 0.006; EO: p = 0.001; ES: *p* = 0.009). There was a significant difference between Ekso-TM and Ekso-OG in ES (*p* = 0.007), but not in RA (*p* = 0.072), or EO (*p* = 0.192), indicating no effect of walking aid on RA or EO EMG amplitude. Of note is that the level of EMG activity elicited by Ekso-assisted walking could be higher than that produced by the MVC trials (Fig. 3; see also Additional file 1: Figure S1).

To determine whether the high amount (50%) of BWS required of the SCI subjects during Loko-TM could account for the low level of EMG activation in that condition, EMG data were recorded from able-bodied participants walking at 50% BWS and near full-weight bearing (5 kg BWS) during Loko-TM (data not shown). Paired t-tests revealed no significant effects of BWS in the able-bodied participants on RA (*p* = 0.58), EO (*p* = 0.49), or ES (*p* = 0.24) activity.

### Breathing

**Fig. 4:**
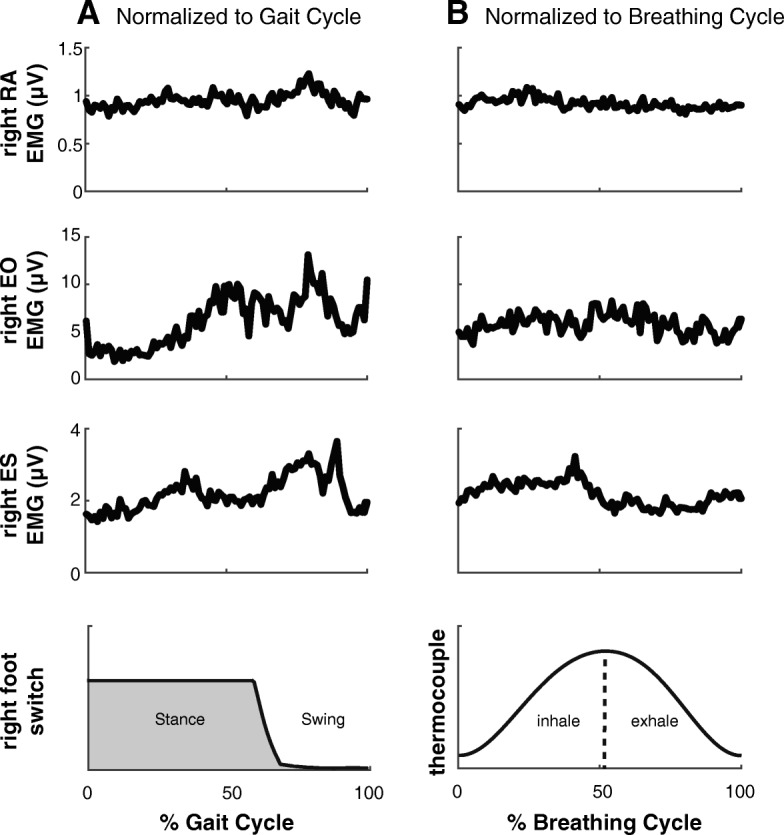
Trunk muscle activity normalized to gait cycle and breathing cycle. SCI subject (S05) average trunk muscles activity (Rectus Abdominis (RA), external oblique (EO) and erector spinae (ES)) normalized to the gait cycle (**a**) vs. normalized to the breathing cycle (**b**) during Ekso-TM

Segregating the trunk muscle EMG to the onset of inspiration revealed no observable pattern of rhythmic activity timed to the respiratory rhythm in any of the muscles. Sample data from an individual SCI subject is shown in Fig. 4, and data from each of the SCI subjects comparing EMG patterns normalized to inhalation vs. right foot contact times are provided in Additional file 2: Figure S2.

### Trunk acceleration

**Fig. 5:**
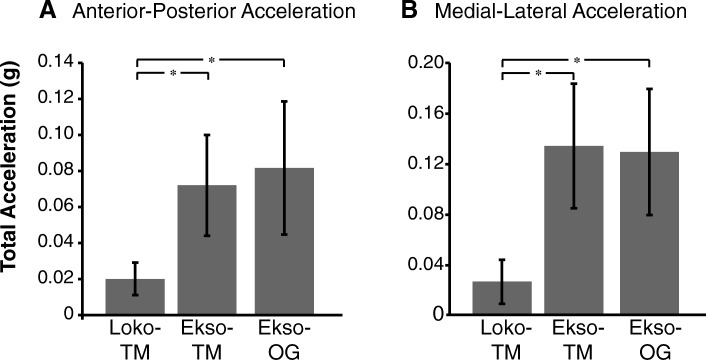
Total acceleration of the trunk across robotic-assisted walking conditions. **a**) Mean total acceleration of the trunk for all SCI subjects across the 3 robotic-assisted walking conditions: Lokomat (Loko-TM), Ekso on treadmill (Ekso-TM) and Ekso overground (Ekso-OG). **b**) Total medial-lateral trunk acceleration during the same walking conditions. * = *p* < 0.001

Figure 5 shows average anterior-posterior and medial-lateral acceleration of SCI subjects during the 3 walking conditions and average able-bodied subjects during regular treadmill walking. There was a main effect of walking condition on the trunk acceleration in both anterior-posterior (F_2,14_ = 15.03, *p* < 0.001, partial η^2^ = 0.68, observed power = 0.99) and medial-lateral directions (F_2,14_ = 17.00, p < 0.001, partial η^2^ = 0.71, observed power = 0.99). Post-hoc analysis showed that anterior-posterior acceleration was significantly lower during Lokomat walking compared to both Ekso-TM (*p* = 0.002) and Ekso-OG (p = 0.002). Medial-lateral acceleration was also significantly lower during Lokomat walking compared to Ekso-TM (*p* = 0.001) and Ekso-OG (p = 0.001). However, there were no statistically significant differences between Ekso-TM and Ekso-OG in either direction (anterior-posterior: *p* = 0.33; medial-lateral: p = 0.33).

## Discussion

In this study we compared trunk muscle activation patterns during walking with a treadmill-based robotic gait training device (Lokomat) and an overground robotic exoskeleton (Ekso) in people with motor-complete SCI. A key finding from this study is that despite the fact that all of the SCI participants enrolled in this study had high-thoracic, motor-complete injuries, we showed that Ekso-assisted walking was effective in activating the trunk muscles, and that the level of activation facilitated was greater than that generated by attempted voluntary contraction of these muscles.

### Ekso is more effective than the Lokomat in engaging the trunk muscles

Although the Lokomat and the Ekso are both used to facilitate gait training in people with SCI, they differ in the way they provide gait training. When used with people with complete SCI, the Lokomat does not require active participation from the user, as the trunk is passively supported by an overhead harness and the legs are moved by the device. In contrast, gait training in the Ekso requires active participation from the user, as they have to shift their body weight from side to side and use a gait aid while maintaining standing balance. It is likely that this alternating weight shifting movement and standing balance required by the Ekso are responsible for the higher activity in the trunk muscles during the Ekso-assisted walking conditions. Aaslund et al. [17] have shown that walking with a weight support harness resulted in restricted trunk acceleration in all directions. Similarly, our acceleration data shows limited trunk movement during the Loko-TM trial and greater trunk acceleration during walking in the Ekso compared to the Lokomat, which is expected due to the weight shifting. In fact, with such restricted trunk movement in the Lokomat, trunk muscle EMG amplitudes (except EO) were no different from that recorded during quiet supine lying.

### Could walking aids have an effect?

In this study, there were two Ekso-assisted walking conditions: Ekso-OG in which the participants walked overground and used a front-wheeled walker, and Ekso-TM in which they walked over a treadmill and used the handrails. Although there were no significant differences in abdominal muscle activity between Ekso-OG and Ekso-TM trials, ES activity was significantly lower in Ekso-TM compared to Ekso-OG. In the Ekso-OG condition, in addition to weight shifting, the participants had to push the wheeled walker with each step, but in the Ekso-TM trial, there is less anterior-posterior acceleration in the trunk likely since forward shifting is facilitated by the movement of the treadmill belt. This lack of forward shifting may have contributed to the lesser ES activation noted in the Ekso-TM trial.

### Patterning of trunk muscle activity

The trunk muscles are normally activated during walking to provide upper body steadiness throughout the gait cycle [9, 10]. Several studies have shown certain patterns of activation for each muscle. In these studies, the abdominal muscles were documented to have variable patterns of activity with most participants exhibiting a low and constant muscle activity throughout the gait cycle. However, some individuals do exhibit rhythmic activation; for instance, RA activity may be rhythmically active with a peak around mid-stance [18] or end-stance [19] while EO may peak close to ipsilateral [19, 20] or contralateral heel strike, especially at higher speeds due to higher postural stability demands [11]. In the paraspinal muscles, activation patterns reported in the literature seem to be more consistent across individuals, with ES rhythmically active and peaking around heel strike [19, 21]. The data from our able-bodied control subjects are consistent with these previous reports [11, 18–22]. Among our able-bodied control group, trunk EMG patterning during walking in the Ekso was similar to that observed during normal overground walking, albeit with higher amplitudes. Sylos-Labinin et al. [23] also observed that able-bodied participants walking with the MINDWALKER research exoskeleton, which has a similar walking mechanism to the Ekso, showed similar or higher trunk muscle activity compared to normal walking. In our SCI subjects, when rhythmic activity was observed, the patterning was qualitatively similar to that reported during normal walking in able-bodied participants [11, 18–22].

### Possible mechanism of trunk muscle activation

As aforementioned, the Ekso requires the user to consciously control their weight-shifting from one side to the other in order to trigger stepping. This voluntary control of shifting the body weight suggests engagement of the cortical and possibly vestibulospinal pathways. Sparing of corticospinal and vestibulospinal inputs to the trunk muscles has been revealed in recent studies [7, 8, 24]. We similarly observed that all SCI subjects were able to elicit activity during the MVC trials. Our data also indicate an ability to modulate this activity according to the postural demands, as supported by the higher trunk muscle activity observed in the Ekso-TM and Ekso-OG walking trials compared to Loko-TM at matched speeds.

It is possible that the muscle activity of the trunk below the level of injury is a result of the spinally mediated activation of stretch reflexes in the abdominal muscles due to the inspiratory activity of the diaphragm and changing intra-abdominal pressure [25] or changes in trunk angle during the gait cycle [26]. In this study, in addition to the controlled breathing protocol followed during the MVC [8], we recorded the breathing pattern during the Ekso and Lokomat assisted walking trials and investigated the patterning of trunk muscle activity with respect to the breathing rhythm. We did not observe any specific trunk muscle activity pattern in relation to the breathing cycle. However, we cannot rule out the possibility that trunk muscle activity could have been facilitated by spindle-mediated stretch reflexes. For instance, Saunders et al. (2005) showed that RA activity during walking in able-bodied subjects is coupled to extension of the trunk. However, even at the slowest speed they tested (1 m/s), which is at least three times faster than walking speeds in our study, the range of motion in the sagittal plane is very low (1.2°) [26]. Thus, it is unlikely that the observed muscle activity in our study was modulated by stretch of the trunk muscles.

### Potential clinical implications and future directions

Seated postural control in SCI is the foundation for many functional activities. Hence, developing effective training strategies is important to prevent performance in daily functional activities and enhance the quality of life for people with SCI. Impaired postural control after SCI occurs as a result of the paralysis or weakness of trunk muscles [4]. As a result, people with SCI are known to develop new postural control synergies by recruiting non-postural control muscles to compensate for the loss of the postural control muscles [27, 28]. However, these new postural control synergies do not fully compensate the function of trunk muscles that are responsible for the normal postural control synergies [29]. Therefore, finding new training strategies to reactivate and train trunk muscles could possibly support normal postural control synergies and ultimately improve seated postural control in people with SCI. Indeed, early results already indicate the benefit of an Ekso-assisted gait training program on seated balance control in people with motor-complete SCI [30].

### Methodological considerations

We were not able to record trunk kinematics or account for the forces exerted by the upper limbs on the walker or siderails during our study, and therefore could not account for the possible effects of these factors on trunk muscle activation. Although, the Ekso-TM condition allowed us to account, to some extent, for differences in upper limb engagement between the Ekso and Lokomat, it is not surprising that the Ekso requires more active engagement of postural muscles since steps are triggered only when the user achieves the desired weight shift target. Our results therefore support the continued development of such rehabilitation devices that could be effective for facilitating trunk muscle activation along with other health benefits in people with motor-complete SCI.

Trunk muscle activation patterns are known to alter with walking speed [11, 12]. Therefore, walking speed had to be matched between the Lokomat and Ekso to allow for an appropriate comparison. The slowest possible treadmill speed with the Lokomat is 1 km/h. Although this walking speed could be considered fast for walking with the Ekso [14, 16, 31], all the participants recruited to this study were able to achieve it.

The Ekso provides full body weight bearing, which is not possible to achieve in the Lokomat with people with high thoracic motor-complete SCI. Trunk muscle activity could be affected by the percentage of the body weight support [32] and the full-weight bearing provided by the Ekso could have contributed to the better engagement of trunk muscles. Due to the level and completeness of injury in our SCI subjects, it was not possible to vary the amount of BWS during Lokomat walking. However, our able-bodied data have shown similar muscle activity during walking with the Lokomat at 50% of BWS compared to 5 kg BWS (the minimum BWS). Therefore, we argue that the higher level of trunk muscle activity in the Ekso-assisted walking conditions compared to the Lokomat was not likely due to the differences in the body weight support requirements between the two devices.

## Conclusions

Recent studies have revealed sparing of trunk muscle function below the injury level [7, 8, 24]. These trunk muscles are important for postural control in sitting and standing positions. In this study, we demonstrated better engagement of the trunk muscles during walking in the Ekso compared to Lokomat. The results of this study raise the possibility for using overground exoskeleton-assisted gait training to help recruit and re-train the trunk musculature in people with motor-complete paralysis in order to help improve postural stability.

## Additional files

Additional file 1: **Figure S1.** Trunk activation patterns in all SCI subjects. Activation patterns recorded from the rectus abdominis (RA), external oblique (EO) and erector spinae (ES) muscles in each SCI subject are plotted separately (individual rows, ordered top to bottom by lesion level), in addition to the ensemble EMG activity in these muscles averaged across all able-bodied subjects and compared across the three walking conditions: Lokomat on treadmill (Loko-TM, black lines), Ekso on treadmill (Ekso-TM, red lines) and Ekso overground (Ekso-OG, blue lines). All data are normalized to 100% of the gait cycle and aligned to right heel contact. Thick vertical bars in each plot represent 50% of the MVC for that muscle. (Data from the left ES were not available from subject S03 due to technical error). (EPS 1582 kb)

Additional file 2: **Figure S2.** Trunk activation patterns normalized to breathing cycle compared to gait cycle. Comparison of EMG recordings from the right rectus abdominis (RA), external oblique (EO) and erector spinae (ES) normalized to the breathing cycle (onset of inspiration, green lines) vs those normalized to the gait cycle (right heel contact, blue lines). Each graph shows the average of at least 50 breath cycles and steps. (EPS 1721 kb)

## Abbreviations

AIS: American Spinal Injury Association Impairment Scale
BAS: Baseline
BWS: Body Weight support
Ekso-OG: Ekso overground
Ekso-TM: Ekso on treadmill
EMG: Electromyography
EO: External oblique
ES: Erector spinae
ISNCSCI: International Standards for Neurological Classification of Spinal Cord Injury
Loko-TM: Lokomat (on treadmill)
MVC: Maximum voluntary contraction
RA: Rectus abdominis
RMS: Root mean squre
SCI: Spinal cord injury
